# Identification of molecules associated with response to abatacept in patients with rheumatoid arthritis

**DOI:** 10.1186/s13075-020-2137-y

**Published:** 2020-03-12

**Authors:** Waka Yokoyama-Kokuryo, Hayato Yamazaki, Tsutomu Takeuchi, Koichi Amano, Jun Kikuchi, Tsuneo Kondo, Seiji Nakamura, Ryoko Sakai, Fumio Hirano, Toshihiro Nanki, Ryuji Koike, Masayoshi Harigai

**Affiliations:** 1 Department of Rheumatology, Tokyo Medical and Dental University, 1-5-45 Yushima, Bunkyo-ku, Tokyo, 113-8519 Japan; 2 Department of Pharmacovigilance, Tokyo Medical and Dental University, 1-5-45 Yushima, Bunkyo-ku, Tokyo, 113-8519 Japan; 3 Department of Rheumatology, Japan Organization of Occupational Health and Safety Chubu Rosai Hospital, 1-10-6 Koumei, Minato-ku, Nagoya-City, Aichi Japan; 4 Division of Rheumatology, Department of Internal Medicine, Keio University School of Medicine, 35 Shinanomachi, Shinjuku-ku, Tokyo, 160-8582 Japan; 5 Department of Rheumatology and Clinical Immunology, Saitama Medical Center, Saitama Medical University, 1981 Kamoda, Kawagoe-shi, Saitama, 350-8550 Japan; 6 DNA Chip Research Inc, 1-15-1 Kaigan, Minato-ku, Tokyo, 105-0022 Japan; 7 Department of Rheumatology, Tokyo Women’s Medical University School of Medicine, 8-1 Kawada-cho, Shinjuku-ku, Tokyo, 162-8666 Japan; 8 Division of Rheumatology, Department of Internal Medicine, Toho University School of Medicine, 6-11-1 Omori-Nishi, Ota-ku, Tokyo, 143-8541 Japan

**Keywords:** Rheumatoid arthritis, Abatacept, Prediction, Microarray, Interferon signature

## Abstract

**Background:**

Abatacept (ABA) is a biological disease-modifying antirheumatic drug (bDMARD) for rheumatoid arthritis (RA). The aim of this study was to identify molecules that are associated with therapeutic responses to ABA in patients with RA.

**Methods:**

Peripheral blood was collected using a PAX gene Blood RNA kit from 45 bDMARD-naïve patients with RA at baseline and at 6 months after the initiation of ABA treatment. Gene expression levels of responders (*n* = 27) and non-responders (*n* = 8) to ABA treatment among patients with RA at baseline were compared using a microarray. The gene expression levels were confirmed using real-time quantitative polymerase chain reaction (RT-qPCR).

**Results:**

Gene expression analysis revealed that the expression levels of 218 genes were significantly higher and those of 392 genes were significantly lower in the responders compared to the non-responders. Gene ontology analysis of the 218 genes identified “response to type I interferon (IFN)” with 24 type I IFN-related genes. RT-qPCR confirmed that there was a strong correlation between the score calculated using the 24 genes and that using *OAS3*, *MX1*, and *IFIT3* (type I IFN score) (rho with the type I IFN score 0.981); the type I IFN score was significantly decreased after treatment with ABA in the responders (*p* < 0.05), but not in the non-responders. The receiver operating characteristic curve analysis of the type I IFN score showed that sensitivity, specificity, and AUC (95% confidence interval) for the responders were 0.82, 1.00, and 0.92 (0.82–1.00), respectively. Further, RT-qPCR demonstrated higher expression levels of *BATF2*, *LAMP3*, *CD83*, *CLEC4A*, *IDO1*, *IRF7*, *STAT1*, *STAT2*, and *TNFSF10* in the responders, all of which are dendritic cell-related genes or type I IFN-related genes with significant biological implications.

**Conclusion:**

Type I IFN score and expression levels of the nine genes may serve as novel biomarkers associated with a clinical response to ABA in patients with RA.

## Background

Rheumatoid arthritis (RA) is characterized by chronic inflammatory polyarthritis, which leads to the destruction of the joints causing pain and disability [1]. Cytotoxic T lymphocyte-associated antigen 4 immunoglobulin fusion protein (CTLA4-Ig, abatacept (ABA)) is a biological disease-modifying antirheumatic drug (bDMARD) for RA. T cells are activated by the interaction of HLA class II molecules on antigen-presenting cells (APCs) with a T cell receptor (TCR) on the surface of T cells in the presence of CD80/86 on APCs and CD28 on T cells. CTLA4-Ig inhibits the activation of T cells by selectively modulating the CD80/86–CD28 interaction [2]. Abatacept is as efficacious as other bDMARDs in terms of clinical, structural, and functional outcomes [3]. In a recent meta-analysis, it was found that the risk of serious infections in humans was lower for treatments using ABA than that using other bDMARDs [4]. The prediction of therapeutic responses to ABA could considerably help identify patients that can benefit from the treatment.

Whole blood transcriptomic profiling using microarrays has been widely used to investigate the action mechanisms and identifying appropriate biomarkers predicting the efficacy or safety of various drugs or treatment. Microarrays have been applied to some bDMARDs including ABA [5–9] to realize precision medicine for RA. Although some promising data have been reported, endeavor to develop novel biomarkers is still required. Here, we report the results of our study to identify molecules associated with therapeutic responses of ABA for patients with RA using a microarray.

## Methods

### Patients

A total of 168 RA patients who fulfilled the 2010 American College of Rheumatology/European League Against Rheumatism classification criteria for RA [10] and who received ABA for the first time were enrolled in this multi-center, prospective cohort study from Keio University, Saitama Medical University and Tokyo Medical and Dental University from June 2010 to December 2012 [11]. Blood samples for the microarray and RT-PCR were collected from 129 of the 168 patients. Forty-five of the 129 patients were bDMARD-naïve, and they were enrolled in this study. All patients had active RA despite the use of conventional synthetic disease-modifying antirheumatic drug (DMARD) for at least 3 months. Treatment efficacy was evaluated using the European League Against Rheumatism (EULAR) response criteria [12]. Patients were observed for 6 months after the initiation of ABA treatment. This study was registered at the University Hospital Medical Information Network Clinical Trials Registry (UMIN000005144). This study was approved by the Ethics Committee of the Tokyo Medical and Dental University Hospital (#836 and #M2015-553-01) and the other participating institutions. All subjects provided written informed consent.

### RNA extraction

Blood from the patients was collected in PAXgene Blood RNA tubes (PreAnalytiX) at baseline and at 6 months after the initiation of ABA treatment. Total RNAs were extracted using PAXgene Blood RNA Kits (PreAnalytiX) following the manufacturer’s instructions. The total RNA quantity and quality were determined using a NanoDrop-1000 spectrophotometer (Thermo Fisher Scientific) and an Agilent 2100 Bioanalyzer (Agilent Technologies).

### Microarray experiment

Cy3-labeled complementary RNAs (cRNAs) were synthesized using Quick Amp Labeling Kits (Agilent). The cRNAs were hybridized at 65 °C for 17 h to Whole Human Genome 44 K Microarrays (Agilent, Design ID: 014850). After washing, the microarrays were scanned using an Agilent DNA microarray scanner (Agilent). The intensity values of each scanned feature were quantified using Agilent Feature Extraction Software (Agilent).

### Microarray data analysis

Signal intensity was adjusted using quantile normalization plus ComBat to reduce the batch effect [13, 14]. After excluding poorly annotated probes and low signal probes (average signal < 100), 10,420 probes were extracted for further statistical analysis. We implemented a functional genomic analysis using the PANTHER Overrepresentation Test. The reference list included all *Homo sapiens* genes, and the annotation dataset was obtained from the GO Ontology database (released November 30, 2016).

### Real-time quantitative polymerase chain reaction analysis

Real-time qPCR (RT-qPCR) analysis was performed using a Custom RT2 Profiler PCR Array (QIAGEN) and RT2 qPCR Primer Assays (QIAGEN) according to the manufacturer’s instructions. cDNA was generated using 400 ng of total RNA. Real-time PCR was performed with a Roche Lightcycler 480 (Roche Diagnostics) using 4 ng cDNA per reaction. The thermal profile was as follows: denaturation (95 °C, 1 min) and amplification (45 cycles; 95 °C, 15 s; 60 °C, 1 min). The second derivative maximum method was used to determine the crossing point (Cp) values. The relative expression of the targeting gene was normalized to 18S rRNA (QIAGEN).

### Statistical analysis

The primary objective of this study was to identify novel molecules associated with therapeutic responses to ABA for patients with RA, and the secondary objective was validation of the results of the previous study [9]. Fisher’s exact test and Student’s *t* test were used to compare the categorical and continuous variables between two groups, respectively. The differences in gene expression at baseline obtained using the microarray and RT-qPCR were analyzed using the Welch’s *t* tests; *p* < 0.05 was considered statistically significant. The type I IFN score was calculated using the *Z*-score methods [15]. Correlation between the IFN signature with 24 genes and that with a smaller number of genes was analyzed by Spearman’s correlation test. The optimal cut-off value for discriminating the responders and non-responders to ABA treatment were determined by receiver operating characteristic curve (ROC) analysis.

## Results

### Clinical characteristics of the patients at baseline

Of the 45 bDMARD-naïve patients with RA from whom blood sample for microarray research was obtained, 27 were classified as good responders (described as responders hereafter, 60.0%); 10, as moderate responders (22.2%); and 8, as non-responders (17.8%) using EULAR response criteria [12]. In order to extract response-associated molecules efficiently, we compared baseline data of the responders and non-responders (Table 1). There was no significant difference in age, sex, prevalence of rheumatoid factor and anti-cyclic citrullinated peptide (CCP) antibody, disease activity, and the use of prednisolone (PSL) between the two groups. For the responder group, the disease duration tended to be longer and methotrexate (MTX) was used more frequently.

**Table 1:** Clinical characteristics of responders and non-responders at baseline

	Responders	Non-responders	*p* value
Number of patients	27	8	
Age, year	59.4 ± 13.1	67.4 ± 12.5	N.S.
Female, *n* (%)	22 (81.5)	5 (62.5)	N.S.
Disease duration, month	109.2 ± 147.9	50.25 ± 56.7	N.S.
RF titer, mg/dl	69.7 ± 78.3(*n* = 26)	83.5 ± 74.3	N.S.
RF positivity, *n* (%)	20 (76.9)	8 (100)	N.S.
Anti-CCP antibody titer, U/ml	92.8 ± 94.0(*n* = 25)	120.7 ± 117	N.S.
Anti-CCP antibody positivity, *n* (%)	25 (91.6)	8 (100)	N.S.
DAS28-CRP	4.37 ± 1.04	3.81 ± 0.98	N.S.
Use of PSL, *n* (%)	5 (18.5)	3 (37.5)	N.S.
PSL dose, mg/day	6.4 ± 5.0	10.25 ± 7.2	N.S.
Use of MTX, *n* (%)	19 (70.0)	2 (25.0)	0.04
MTX dose, mg/week	10.61 ± 3.7	9.0 ± 4.2	N.S.

Values are expressed as the mean ± SD. Fisher’s exact test and Student’s *t* test were used to compare categorical and continuous variables between the two groups, respectively. *p* < 0.05 was considered statistically significant

*N.S.* not significant, *RF* rheumatoid factor, *CCP* cyclic citrullinated peptide, *DAS28-CRP* disease activity score in 28 joints using C-reactive protein, *PSL* prednisolone, *MTX* methotrexate

### Genes associated with clinical response to ABA treatment

To identify novel biomarkers associated with clinical responses to ABA treatment, we compared gene expression levels at baseline between the responders and the non-responders. The expression levels of 218 genes in the responders was significantly higher than that of the non-responders, and the expression levels of 392 genes in the responders was significantly lower than that of the non-responders (*p* < 0.05, false discovery rate (FDR) < 0.333 and fold change > 1.3) (Supplementary data 1). Gene ontology (GO) analysis of the 218 genes identified “response to type I interferon (IFN) (GO:0034340)” with 24 type I IFN-related genes: *BST2*, *GBP2*, *IFI27*, *IFI35*, *IFI6*, *IFIT1*, *IFIT2*, *IFIT3*, *IFITM1*, *IFITM3*, *IRF7, ISG15*, *ISG20*, *MX1*, *MX2*, *OAS1*, *OAS2*, *OAS3*, *OASL*, *RSAD2*, *STAT1*, *STAT*2, *TRIM56*, and *XAF1* [16]. Twelve out of the 24 type I IFN-related genes were elevated (*p* < 0.05 without conditions of FDR or fold changes) in the responders compared to the moderate responders plus non-responders (*n* = 18) (Supplementary Table 1 and Supplementary data 2) and the GO analysis again identified “response to type I interferon (IFN).” The GO analysis of the 392 genes downregulated in the responders did not identify a specific group of genes. The previously reported genes associated with therapeutic response to ABA, which were elongation arrest and recovery-related genes and CD56-specifically expressed genes [9], were not included in the over- or under-expressed genes.

### Type I IFN score and treatment response to ABA

**Fig. 1:**
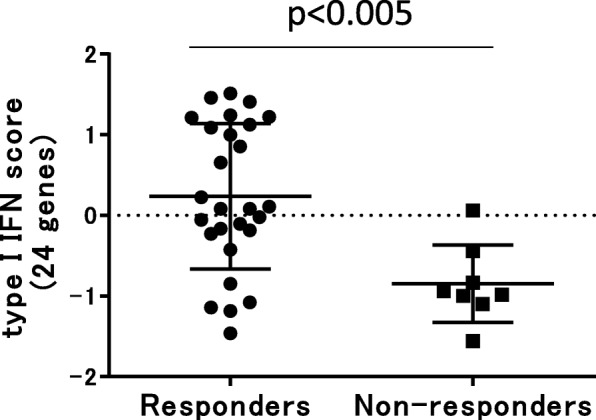
Comparison of type I IFN scores between responders and non-responders. Type I IFN score was calculated using the average values of the *Z*-scored 24 type I IFN genes, as reported by Kennedy et al. [15]. Responders to abatacept showed higher type I IFN score than non-responders (*p* < 0.005, the Mann-Whitney’s *U* test)

To evaluate the association of the type I IFN signature and treatment response to ABA, we calculated the type I IFN score using the average values of the *Z*-scored 24 type I IFN genes, as reported by Kennedy et al. [15]. The type I IFN score of the responders was significantly higher than the non-responders (*p* < 0.005, Fig. 1). In order to reproduce the type I IFN score with fewer genes, we compared the type I IFN score calculated using the 24 genes and the scores created by a combination of some of the genes (Supplementary Fig. 1A). We found that there was a strong correlation between the scores calculated by the 24 genes and the score created using genes of *OAS3*, *MX1*, and *IFIT3* (rho with the type I IFN score 0.981) (designated as type I IFN score hereafter) (Supplementary Fig. 1B).

**Fig. 2:**
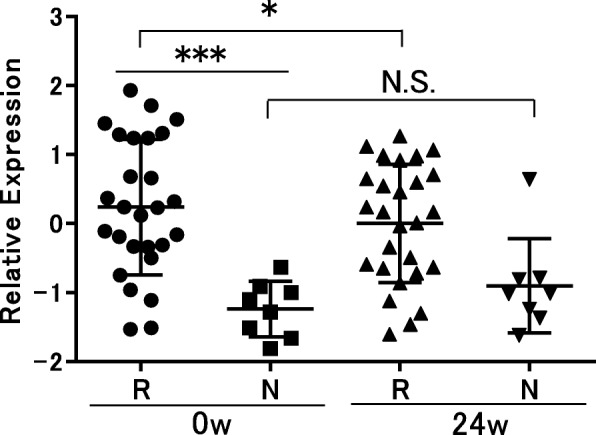
Type I IFN score using RT-qPCR at baseline and 24 weeks after the initiation of abatacept treatment. The expression levels of *OAS3*, *MX1*, and *IFIT3* were determined by using RT-qPCR to calculate the type I IFN score for the same RNA samples of microarray analysis (Fig. 2). *p* < 0.05 was considered statistically significant. *p < 0.05, **p < 0.01, ***p < 0.001. R, responders; N, non-responders; N.S. not significant

To confirm the expression levels of genes using the microarray analysis and their association with the treatment response to ABA, we performed RT-qPCR; we quantified the expression levels of *OAS3*, *MX1*, and *IFIT3* to calculate the type I IFN score using the same RNA samples used for microarray analysis. The type I IFN score using RT-qPCR of the responders was significantly higher than that of the non-responders (*p* < 0.0005, Fig. 2). We also compared the type I IFN score at baseline and at 24 weeks after the initiation of ABA treatment. The type I IFN score using RT-qPCR significantly decreased, albeit only a 15% reduction, after treatment with ABA in the responders (*p* < 0.05, Fig. 2); however, this was not observed for the non-responders.

The ROC analysis revealed an optima cutoff value of the relative expression levels as − 0.565. Sensitivity, specificity, and AUC (95% confidence interval) were 0.82, 1.00, and 0.92 (0.82–1.00), respectively (Supplementary Fig. 2).

### Other treatment response-associated molecules confirmed by RT-qPCR

**Fig. 3:**
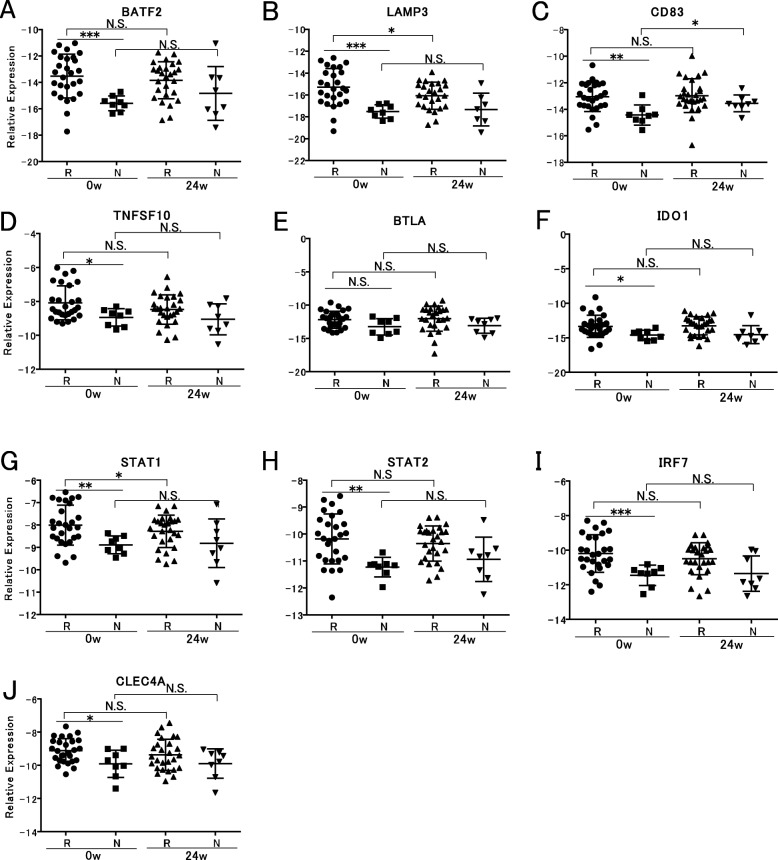
Comparison of mRNA expression levels of the selected genes at baseline between the responders and the non-responders. Expression levels of *BATF2*, *LAMP3*, *CD83*, *TNFSF10*, *BTLA*, *CLEC4A*, *IDO1*, *STAT1*, *STAT2*, and *IRF7* were determined using RT-qPCR and compared between the responders and the non-responders (**a**–**j**). *p* < 0.05 was considered statistically significant. *p < 0.05, **p < 0.01, ***p < 0.001. R, responders; N, non-responders, N.S., not significant

Since type I IFN is primarily produced by plasmacytoid dendritic cells (pDC), we selected dendritic cell-related genes or type I IFN-related genes with significant biological implications for quantification using RT-qPCR among the 218 genes as follows: *BATF2*, *LAMP3*, and *CD83* are related to dendritic cell activation and maturation [17–19]; *TNFSF10*, *BTLA*, and IDO1 are expressed on dendritic cells (DCs) [20–24]. *CLEC4A* has a role in the production of type I IFN from pDC [25], and *STAT1*, *STAT2*, and *IRF7* have roles in the signal of type I IFN production [26–28]. The expression levels of these 10 genes measured by qRT-PCR in the responders were significantly higher compared to those of the non-responders except for *BTLA* (Fig. 3). We compared gene expression levels among patients with different disease activities at baseline and 24 weeks after the initiation of ABA treatment, and it found that all genes had no association with the disease activities at both time points (data not shown).

We compared the expression levels of these 10 genes before and after treatment with ABA using RT-qPCR. The expressions of *LAMP3* and *STAT1* were significantly decreased after treatment with ABA; however, the percentage of reduction was relatively small (*LAMP* 41.7% and *STAT1* 17.4%, Fig. 3b, g).

## Discussion

In this study, we demonstrated that the type I IFN score and the expression levels of *BATF2*, *LAMP3*, *CD83*, *CLEC4A*, *IDO1*, *IRF7*, *STAT1*, *STAT2*, and *TNFSF10* are associated with a good clinical response to ABA in patients with RA.

The family of type I IFNs, which consist of IFN-alpha and IFN-beta, has an important role in regulating immune response [28] [29]. The high expression of the type I IFN signature was found in 22 to 65% of the patients with RA [27, 30] but was not associated with disease activity [31]. It has been reported that the type I IFN signature is highly expressed in the pre-clinical phase of RA with increased levels of anti-CCP antibody and rheumatoid factor [32, 33]. In addition, IFN-administered patients often develop arthritis as an adverse drug reaction [34–36], which indicates that the increased levels of type I IFN, triggered by a viral infection or other immunological stimuli, may be involved in the pathogenesis of pre-clinical or early RA. It is reported that arthritis was mitigated in interferon alpha/beta receptor alpha chain-deficient mice and interferon regulatory factor-1-deficient mice [37, 38]. These reports together with our data may indicate that ABA shows its clinical efficacy through the reduction of the type I IFN activity in patients with RA.

The expression levels of genes related to the activation of dendritic cells, *BATF2*, *LAMP3*, and *CD83*, showed significant differences between responders and non-responders at baseline. *LAMP3* was one of the differentially expressed genes between RA and osteoarthritis patients [39], and *CD83* was expressed in more than 20% of pDCs in the RA synovium [40]. In addition, early-stage RA patients had elevated levels of soluble *CD83* in plasma [41]. Since *CD83* is expressed as a membrane-bound form on mature dendritic cells and as a soluble form in plasma, further studies are warranted to evaluate the predictive ability of *CD83* mRNA or proteins for responses to ABA treatment or to other treatments in patients with RA.

Comparing the background at the start of ABA treatment, the percentage of MTX users was different in responders and non-responders. Recently, it has been reported that the expression level of the type 1 IFN is higher in patients that do not respond to methotrexate [42]. As there was no difference in the type I IFN scores among MTX users and non-users in both responders and non-responders in this study (data not shown), the cause of the difference in type I IFN expressions between the responders and the non-responders is not attributed to the percentage of MTX use.

This study has some limitations. First is the small sample size. The association of IFN signature with therapeutic response to ABA identified between responders (*n* = 27) and non-responders (*n* = 8) was supported by the comparison between the responders vs moderate- plus no-responders (*n* = 18). Second, we did not have validation cohort, and the risk of over-fitting of models should be considered. Our results need to be confirmed in a future study. Third, we could not validate the results of the previous study, in which the signature scores of elongation arrest and recovery-related genes, and CD56-specifically expressed genes were significantly elevated in non-responders [9]. The characteristics of the patient population analyzed and the definition of therapeutic response applied may account for the difference between the studies.

## Conclusion

Type I IFN score and expression levels of the nine genes—*BATF2*, *LAMP3*, *CD83*, *TNFSF10*, *CLEC4A*, *IDO1*, *STAT1*, *STAT2*, and *IRF7*—may serve as biomarkers for predicting the clinical responses to ABA treatment in patients with RA.

## Supplementary information

**Additional file 1: Figure S1A, B**. Correlation between the IFN signature with 24 genes and the IFN signature with a smaller number of genes from the 24 genes. **Figure S2**. The receiver operating characteristics curve of the type I IFN score for the responders. (PPTX 65 kb)

**Additional file 2: Table S1**. Clinical characteristics of EULAR responders vs moderate and non-responders at baseline.

**Additional file 3.** List of genes with higher and lower expression levels in EULAR good responders compared to non-responders.

**Additional file 4.** List of genes with higher and lower expression levels in EULAR good responders compared to moderate and non-responders.

## Data Availability

The datasets generated and/or analyzed during the current study are not publicly available due to future analysis plans but are available upon request under the condition of collaboration.

## Abbreviations

ABA: Abatacept
APCs: Antigen-presenting cells
bDMARDs: Biological disease-modifying antirheumatic drugs
CCP: Cyclic citrullinated peptide
csDMARD: Conventional synthetic DMARD
DMARD: Disease-modifying antirheumatic drug
EULAR: The European League Against Rheumatism
FDR: False discovery rate
GO: Gene ontology
IFN: Interferon
IFX: Infliximab
MTX: Methotrexate
PSL: Prednisolone
RA: Rheumatoid arthritis
RT-qPCR: Real-time quantitative polymerase chain reaction
RTX: Rituximab
TCR: T cell receptor
