# Supplementary material for: Identification of molecules associated with response to abatacept in patients with rheumatoid arthritis
Source: Arthritis Res Ther. 2020 Mar 12;22:46. doi: 10.1186/s13075-020-2137-y (PMC7068901; doi:10.1186/s13075-020-2137-y)
Supplement: Supplementary file 1 — Additional file 1: Figure S1A, B. Correlation between the IFN signature with 24 genes and the IFN signature with a smaller number of genes from the 24 genes. Figure S2. The receiver operating characteristics curve of the type I IFN score for the responders. (PPTX 65 kb) [file 13075_2020_2137_MOESM1_ESM.pptx]

## Slide 1
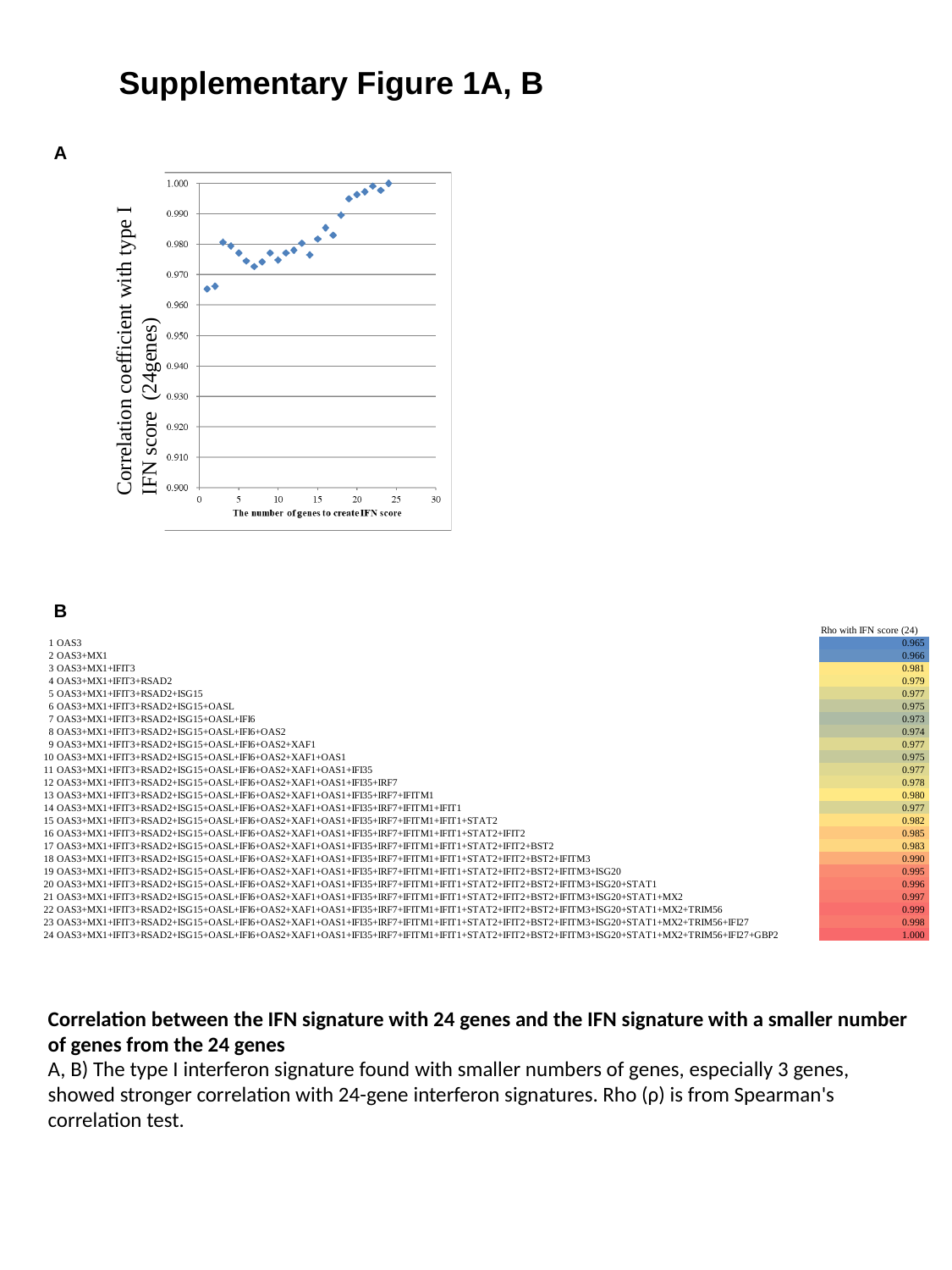

Supplementary Figure 1A, B
　　A
Correlation coefficient with type I IFN score (24genes)
　　B
Correlation between the IFN signature with 24 genes and the IFN signature with a smaller number of genes from the 24 genes
A, B) The type I interferon signature found with smaller numbers of genes, especially 3 genes, showed stronger correlation with 24-gene interferon signatures. Rho (ρ) is from Spearman's correlation test.

## Slide 2
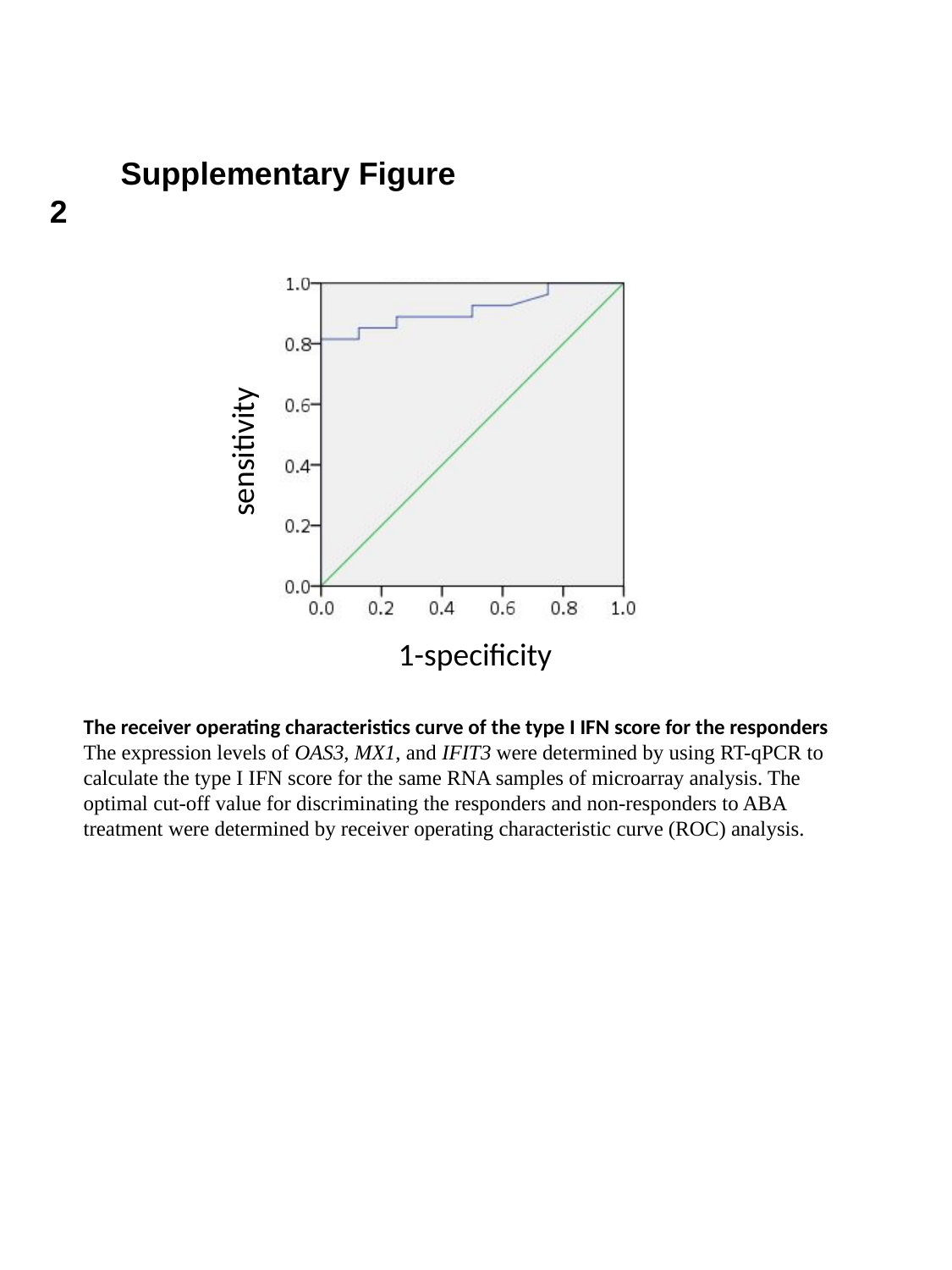

Supplementary Figure 2
sensitivity
1-specificity
The receiver operating characteristics curve of the type I IFN score for the responders
The expression levels of OAS3, MX1, and IFIT3 were determined by using RT-qPCR to calculate the type I IFN score for the same RNA samples of microarray analysis. The optimal cut-off value for discriminating the responders and non-responders to ABA treatment were determined by receiver operating characteristic curve (ROC) analysis.
