# Supplementary material for: Identification of molecules associated with response to abatacept in patients with rheumatoid arthritis
Source: Arthritis Res Ther. 2020 Mar 12;22:46. doi: 10.1186/s13075-020-2137-y (PMC7068901; doi:10.1186/s13075-020-2137-y)
Supplement: Supplementary file 2 — Additional file 2: Table S1. Clinical characteristics of EULAR responders vs moderate and non-responders at baseline. [file 13075_2020_2137_MOESM2_ESM.docx]

|  | Responders | Moderate responders plus non-responders | P‐value |
| --- | --- | --- | --- |
| Number of patients | 27 | 18 |  |
| Age, year | 59.4 ± 13.1 | 68.0 ± 9.65 | 0.01 |
| Female, n (%) | 22 (81.5) | 14 (77.7) | N.S |
| Disease duration, month | 109.2 ± 147.9 | 72.9 ± 94.8 | N.S |
| RF titer, mg/dl  RF positivity, n (%) | 69.7 ± 78.3（n = 26）  20 (76.9) | 110.5 ± 182.3  15 (83.3) | N.S  N.S |
| Anti CCP antibody titer, U/ml | 92.8 ± 94.0（n = 25） | 98.21 ± 100.5 | N.S |
| Anti CCP antibody positivity, n (%) | 25 (91.6) | 16 (88.9) | N.S |
| DAS28-CRP | 4.37 ± 1.04 | 3.93 ± 1.33 | N.S |
| Use of PSL, n (%) | 5 (18.5) | 8 (44.4) | N.S |
| PSL dose, mg/day | 6.4 ± 5.0 | 5.53 ± 5.48 | N.S |
| Use of MTX, n (%) | 19 (70.0) | 11 (61.1) | N.S |
| MTX dose mg/week | 10.61 ± 3.7 | 8.00 ± 2.83 | 0.04 |

**Supplementary Table. Clinical characteristics of EULAR responders vs moderate and non-responders at baseline**

Values are expressed as the mean ± SD. Fisher’s exact test and Student’s t-test were used to compare categorical and continuous variables between the two groups, respectively. P<0.05 was considered statistically significant. N.S. = not significant.

RF = rheumatoid factor, CCP = cyclic citrullinated peptide, DAS28-CRP = disease activity score in 28 joints using C-reactive protein, PSL = prednisolone, MTX = methotrexate
